# Report from the Medical Wards of the Illinois General Hospital

**Published:** 1853-09

**Authors:** N. S. Davis

**Affiliations:** One of the Physicians to the Hospital, and Prof. of Pathology, Practice of Medicine, and Clinical Medicine in Rush Medical College


ART. IV.—*Report from the Medical Wards of the Illinois General Hospital.* By N. S. Davis, M.D., one of the Physicians to the Hospital, and Prof. of Pathology, Practice of Medicine, and Clinical Medicine in Rush Medical College.

During the seven months intervening from January 1st, 1853, to August 1st, the whole number of Cases treated in the *Medical* Wards of the hospital were 150; of which 72 were acute diseases, and the remainder chronic affections. Of those classed as *acute*, 18 are registered under the head of Fever, 8 under that of Dysentery, 6 under that of Cholera Morbus or Diarrhœa with Vomiting, 7 of Acute Opthalmia, 6 of Delirium Tremens, 5 of Acute Rheumatism, 11 of Pneumonia, 4 of Pleurisy, 1 of Pericarditis, 1 of Œdema of the lungs, 2 of Erysipelas, and 3 died within the first twenty-four hours after admission, being too near dead when brought in, to admit of either diagnosis or treatment. Of the 69 cases of acute disease treated, 8 proved fatal, as follows, viz.: of Fever 2, Dysentery 2, Pneumonia 3, and Œdema of the Lungs 1.

The two fatal cases of fever were complicated with severe local diseases. The first was a female, aged about 35 years, Norwegian by birth, and the mother of one or more children. She was admitted into the hospital January 4th; says she has had Intermittent fever since October previous. Present appearance and symptoms: face, lips, and tongue pale and thin, considerable general emaciation, pulse 100 per minute, soft and small; tongue clean, bowels regular, respiration rather short and hurried, and the spleen so much enlarged as to fill the whole left hypochondriac region, and extend downward to the crest of the illium; the tumor was not painful and only slightly tender to the touch. The appearance of the patient was that of great anæmia or deficiency of red corpuscles in the blood, but without Œdema of the extremities, or appreciable effusion into any of the serous cavities. She is still in the hospital, having a chill followed by a short paroxysm of febrile excitement every day. The sweating stage, however, is very slight or altogether wanting. She was ordered the following powder, to be taken every four hours, viz.:

Chloride of Sodium, 10 grs.
Sulph. Quinine, 3 grs.
Pulv. Opii, 1 gr., mix. Also diet of animal broth. She continued this treatment until the morning of the 6th, without any recurrence of the chills, and with an evident improvement in all the symptoms. This morning, however, she complains of some pain in the left side with more tenderness over the enlarged spleen.

Having had no movement of the bowels during the previous 48 hours, she was ordered three powders, each composed of Pulv. Doveri, 5 grs., and Sub. Murias Hydrarg, 2 grs., to be taken one every four hours, and the whole followed by a laxative of castor oil. But before all the powders had been taken, copious dark fœcal evacuations occurred, and the oil was omitted. On the morning of the 7th, she had no pain or tenderness in the left side and the spleen was considerably reduced in size. The bowels continued to move often, the discharges being liquid and dark brown color, with much general debility. She was ordered a tea-spoonful of the following emulsion every four hours, with 2 grains of Sulphate of Quinine in each dose, viz.:

℞ Oil Turpentine ʒjj.

Tinct, Opii, ʒjj.

Gum Arabic, } aa ʒjjj. Rub together thor-

White Sugar, }

oughly and add water ℥jj, mix.

She continued this until the morning of the 10th, when the bowels being less relaxed, the spleen rapidly diminishing in size, and no return of chills, she was directed the same medicine only three times per day. This she continued until the morning of the 13th, when the enlargement of the spleen was found to have entirely disappeared; but there remained a tender irritable state of the mucous membrane of the illium, characterized by two or three evacuations per day of a semi-fluid consistence and brownish color; no appetite; skin dry; and the debility with the anæmic appearances as strongly marked as at the beginning. Wishing to counteract the anæmia by improving the quality of the blood, she was ordered the Persesqui nitrate of iron, in doses of 7 drops, four times per day, with a diet of boiled milk and flour. The next morning, however, finding the iron not well borne by the stomach, she was directed the following powder, to be repeated every six hours, viz.:

| | | |
|---|---|---|
| ℞ | Chloride of Sodium, | 10 grs. |
| | Sulph. Quinine, | 2 grs. |
| | Carb. Iron, | 4 grs. |
| | Pulv. Opii, | ½ gr.—mix. |

On the following morning (the 15th), the looseness of the bowels was found to have greatly increased, accompanied by considerable griping pain, and she was directed Pulv. Opii 1 gr., Acetas Plumbi 2 grs., and Sulph. Quinine 1 gr., mixed, every four hours, with beef-tea. This was continued until the morning of the 18th, with an abatement but not suppression of the diarrhœa. In the mean time the urine had become very scanty and high colored, and a slight appearance of œdema of the extremities. Wishing to avoid the effects of opium on the kidneys, she was directed to omit the powders, and take a tea-spoon full of the emulsion of oil of turpentine and laudanum every two hours, with an infusion of juniper berries and uva ursi for a drink. This treatment was continued until the 25th, with no other apparent effect than to restrain the bowels, increase moderately the action of the kidneys, and palliate the general symptoms. But this morning the mucous membranes are again more irritable and the symptoms of anæmia with œdema of the extremities slowly increasing. Contrary to directions she eat yesterday some fat broiled pork, which undoubtedly increased the gastric disturbance. She was directed to continue the emulsion with ¼ gr. of nitrate of silver each morning and evening in the form of pill.

Under this treatment the condition of the mucous membranes improved slowly until the 29th, but the general anœmic condition and debility had rather increased. The food taken did not seem to replenish the blood or nourish the tissues.

She was again ordered the persesqui nitrate of iron in doses of 8 drops every 8 hours, with a tea-spoonful of the emulsion between each dose. Finding no improvement, on the first day of February, she was allowed, in addition, (at her own request) a glass of Porter three times a day. From this time the discharges from the bowels became more frequent, the urine more scanty, the œdema of the cellular tissue greater, and some cough with bronchial rales. She was directed at different times small doses of sulphas. cupri with opium, nitrate of bismuth, anodyne and astringent injections, and brandy punch, but all without benefit, and she died exhausted on the 10th of February, thirty-seven days after admission. At the close of this case we find the following note in the memorandum book kept by Dr. H. Parker, then assistant physician in the hospital, viz.:

"Feb. 10th, patient died at 7 o'clock, A. M. The discharges from the bowels continued while life lasted, nothing seeming to control them. The functions of assimilation and nutrition seem to have been almost entirely suspended from the date of her admission; the blood becoming so impoverished and thin as to permeate its own vessels, favored also by a want of tonicity in the vessels themselves. The spleen had become reduced to its normal size since the second week after her admission, and the liver not being enlarged, I infer there was no obstruction of the portal circulation. What, then, was the cause of the œdema, except the impaired condition of the blood and the vessels containing it?"

*Remarks:* The points particularly worthy of attention in this case are, 1st, the direct effects of the periodical fever on the functions of assimilation and nutrition including the blood; 2nd, the prompt subsidence of the splenitic enlargement after the paroxysms of ague were arrested; and 3d, the subsequent persistent diarrhœa, anæmia and dropsical effusion.

The second fatal case of fever, was a German aged sixty years, admitted into the hospital April 15th, in a state of extreme prostration. His tongue was dry and brown, eyes heavy and sunken, pulse very small and feeble, chest dull on both sides, breathing laborious, with cough and bloody expectoration. His intellect was extremely dull, it being difficult to arouse him sufficiently to elicit intelligent answers. He had the appearance of having been sick with typhoid fever, complicated with pneumonia, for three weeks. He died on the 7th, about 48 hours after admission.

Of the 16 cases of fever that recovered, four are registered as of the *periodical*, and twelve of the *continued* type.

It must be borne in mind that the period of time embraced in this report includes six of the healthiest months in the year, those in which there is especially the least prevalence of fevers. Three of the four cases registered as periodical were of the intermittent form; and all of them chronic or relapsing cases, accompanied by an impoverished condition of the blood and a relaxed or impaired state of the solid tissues. The recurrence of the chills and febrile exacerbations was generally effectually interrupted within the first 48 hours by the exhibition of chloride of sodium and sulph. quinine in the proportion of 15 grs. of the former to 3 grs. of the latter, repeated every four or six hours; after which a continuance of the same medicine with the addition of three grains of phosphate of iron, and repeated morning and evening, with a plain but nutritious diet, restored the blood and solids to their natural condition in from one to three weeks. If there was any tendency to diarrhœa opium, in doses of one or two grains, was added to the foregoing medicine, or as a substitute for the whole, the patients were directed the emulsion of oil of turpentine and Tinct. Opii with from two to four grains of Quinine added to each dose. Ten of the twelve cases entered under the head of *continued fever* presented well marked symptoms of intestinal irritation. They were all brought to the hospital between the end of the first and the commencement of the third week after the disease had fairly commenced. Generally they presented a dull, heavy, stupid expression of countenance; skin moderately hot and dry, with a feeling of roughness; mouth and tongue dry, the latter sometimes red and smooth, at others covered with a dark brown coat; abdomen moderately tympanitic and tender to firm pressure; pulse varying from 90 to 120 per minute, soft and easily compressed; capillary circulation in the surface slow and feeble; discharges from the bowels from three to six per day, and almost always dark brown and liquid; and the urine scanty and high colored. In only three of the cases was there any eruption over the abdomen and chest. In four there was dullness on percussion over the inferior and posterior lobes of the lungs, with some cough and shortness of breath. In nearly all the cases delirium of a low, wandering character was present during some part of their course, and dulness of hearing was a common symptom. By technical writers they would be called *typhoid* cases; while the remaining two being destitute of symptoms of intestinal irritation would be classed as *typhus*. A large proportion of them had taken mercurials and purgatives, and some of them liberal doses of quinine and opium before their admission to the hospital. Owing to these facts it has been found impossible to adopt any system of treatment applicable indiscriminately to all cases, more especially as the local complications often demand more attention than the primary general disease. It is indeed very rare that a case of continued fever reaches the hospital in its first stage, or during that period when even Dr. Fenner would consider his *abortive* treatment by large doses of quinine applicable. As the cases present themselves in our wards, we have invariably found the constituents of the blood altered in their qualities, the capillary circulation and secretions diminished, and the elementary susceptibility of all the solids impaired. The evidences of the first are the dark color of the blood when drawn, the slow coagulation of its fibrin, and the altered appearance of its corpuscles under the microscope; of the second, are the dryness of the skin, mouth, and bronchial membrane, and the diminished secretion from the kidneys; while the third is indicated equally plain by the torpid or impaired condition of the capillary vessels, the slow, hesitating action of the voluntary muscles, the mental dulness and indifference, &c. This we consider the essential part of the disease—the general typhoid condition. Superadded to this general disease, we have in a large majority of cases unmistakeable evidences of intestinal disease, tending rapidly to ulcerations in the mucous membrane of the ilium and enlargement of the mesenteric glands; while in a much smaller proportion of cases a low, or rather congestive, grade of bronchial or pneumonic inflammation occurs; and in a still smaller proportion of cases the same grade of inflammatory action occurs in the brain and its membranes. No evidence of the latter disease was present in any of the cases included in this report; the passive delirium and mental dulness being attributable to a state very different, if not the exact reverse, from inflammation. In one or two of the cases, the local complications were slight, leaving the general typhoid condition the principal object of the treatment. With these, rest, a diet of beef tea pretty well salted, two or three small doses of blue mass, Dover's powder, and soda, followed by a laxative dose of castor oil and oil of turpentine; and the subsequent exhibition of chloride of sodium 10 grs. to 20 grs., combined with from half a grain to one grain of opium every 4 or six hours, has been sufficient to restore the blood and the organic susceptibility to their normal condition, and establish convalescence in from four to ten days after admission.

As already remarked, however, in a large majority of the cases the intestinal complication was prominent and severe. The following case will serve as an example of this variety of the disease, with the treatment.

(*Report Continued next month.*)

